# In-Line Enrichment of Cancer Cells from Whole Blood by Cell Self-Organization in Acoustic Fields

**DOI:** 10.1021/acs.analchem.5c01459

**Published:** 2025-06-14

**Authors:** Richard Soller, Ola Jakobsson, Per Augustsson

**Affiliations:** Department of Biomedical Engineering, 5193 Lund University, Lund 223 63, Sweden

## Abstract

We present a novel method for in-line acoustofluidic separation of cancer cells directly from whole blood. We report for the first time the observation that blood cells can self-organize when packed closely together in an acoustic field. We utilize this effect to enrich K-562 cancer cells and white blood cell subpopulations from whole blood at normal hematocrit levels. Further, we investigate the effect of self-organization of particles and cells with different sizes and acoustic properties. We propose that the mechanism for the phenomenon is based on the change of sign of the acoustic contrast of cancer cells relative to the densely packed red blood cells such that they are pushed to the RBC–plasma interface. This method may be useful as a rapid and gentle initial refinement stage for accessing rare cells, potentially enhancing point-of-care diagnostics and advancing treatments like immunotherapy.

## Graphical abstract

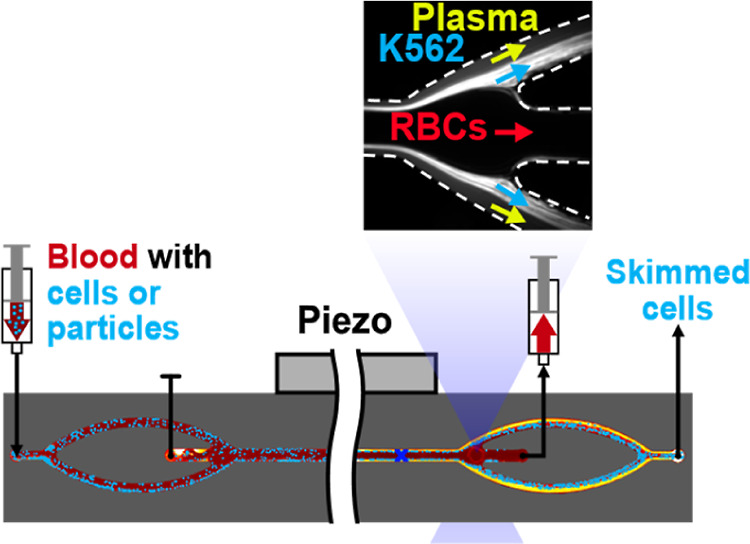

## Introduction

Blood is the major carrier of nutrients, waste products, and chemically encoded information in our body, and it is central to our immune system and is therefore an important source of information about our health. Blood tests, such as hematocrit (HCT) and hemoglobin levels, genome sequencing,^1–3^ cytokine release measurements,^4–6^ white blood cell counts, blood gas, and infection parameters are routinely guiding healthcare professionals in treatment decisions. To meet demands for a shorter time from sampling to result, there is an increased interest to shift from analysis in centralized laboratories to point-of-care tests or patient self-testing.^7^ Point-of-care equipment must be autonomous and robust to enable measurements by nonspecialist operators^8^ and achieve this with a reasonable price and size of equipment. Beyond routine diagnostics, advances in blood analysis have enabled new approaches to disease detection or monitoring. Circulating tumor cell (CTC) isolation, for example, has emerged as a promising tool to monitor and understand metastasizing cancer. CTCs are rare cells shed from primary tumors into the bloodstream, and their isolation and analysis can provide critical insights into cancer progression, metastasis, and treatment response.^9^ Besides its diagnostic use, current breakthroughs in advanced immunotherapy rely heavily on the precise and gentle separation of blood. One example is cancer immunotherapy, where T-cells are isolated from blood and are genetically reprogrammed so that they can be infused back into the patient where they will seek out and destroy cancer cells.^10^ It is an important challenge to make these currently complex and expensive treatments available at local hospitals around the globe. This necessitates automated equipment that can perform a sequence of rare-cell purification steps and exposure of viable cells to reagents using undiluted blood as starting material.

The high precision and flexibility for multistage serial processing offered in microfluidic systems open ways for fast and automated isolation of rare cell populations, such as white blood cell (WBC) subpopulations,^11,12^ and circulating tumor cells.^13–16^ Blood is, however, an extremely diverse and crowded cell suspension. About 40% of the blood volume consists of red blood cells (RBC), which makes it impossible to isolate rare species directly from undiluted blood by most available microfluidic methods with only a few exceptions. Microfiltration, where cells are separated by their ability to squeeze through fine pores^17^ and deterministic lateral displacement,^18^ where cells are separated by their different sideways displacement in a post array, has been demonstrated for purging of RBCs from undiluted whole blood for the purpose of CTC enrichment.^13,17^ Drawbacks with these techniques are problems with cell rupture and consequent fouling due to the frequent interaction of cells with the internal walls of the structure and, for deterministic lateral displacement, challenges related to the fabrication of aspect ratio structures with precise array spacing.^19^

Acoustofluidics, wherein sound scattering forces are exploited to separate cells, has been demonstrated for blood cell fractionation in many-fold diluted blood^20,21^ or for nucleated cells remaining after RBC-lysis.^22–26^ However, previous work on acoustofluidic blood separation has mainly been limited to time-of-flight separation in hundred-fold diluted blood. In this regime, the force on a cell stems from scattered sound due to the differences in density and compressibility of the cell relative to the surrounding, diluted, blood plasma. It has proven challenging to increase the cell concentration beyond a few million cells per ml due to hydrodynamic coupling between closely spaced cells,^27^ which causes them to move as a group rather than based on their individual properties, and has thus limited the applications to platelet-rich blood plasma generation^28–30^ and HCT determination.^31^ Recently, it has been demonstrated that cultured cancer cells, due to their large size compared to blood cells, can be isolated directly from whole blood by acoustically generated microstreaming, although thus far only at 12 μL min^–1^ flow rate.^32^

In this work, we present for the first time a mode of separation that exploits a regime where cells are brought into a state of full contact by means of acoustic focusing, and we study their reorganization relative to each other. Our main findings are (1) that cells reorganize by a mechanism wherein cells that are less dense or more compressible than RBCs are pushed out toward the RBC–plasma interface, and (2) that this reorganization can be exploited to enrich rare cells directly from whole blood in a continuous process. We envision that this type of separation can form an initial refinement stage in a series of purification steps for accessing rare cells such as WBCs or CTCs.

## Theory

Sound can generate acoustic radiation forces on cells suspended in a liquid within a microchannel. Vibrations from a piezoelectric actuator induce a standing pressure half-wave across the width (*w*) of a fluid-filled microchannel if the frequency (*f*) fulfills the condition *f* = *c*/(2*w*) where *c* is the speed of sound in the fluid. The resulting wave, with acoustic energy density (*E*_ac_) and wave vector (*k*_*y*_), scatters on the cells in the liquid, resulting in an acoustic radiation force (***F***_r_) on a cell. This force depends on the cell diameter (*a*) and its acoustic contrast factor (Φ), which derives from the cell’s density (ρ_c_) and compressibility (κ_c_) relative to its surrounding medium density (ρ_m_) and compressibility (κ_m_). For a channel with stiff walls at *y* = −*w*/2 and *y* = *w*/2, ***F***_r_ and the resulting velocity (***u***_r_), in a fluid of viscosity (η), can be expressed as

$$F_{r}=-4\pi a^{3}\Phi E_{ac}k_{y}\sin(2k_{y})e_{y}$$ 1A

$$u_{r}=-\frac{2}{3\eta }a^{2}\Phi E_{ac}k_{y}\sin(2k_{y})e_{y}$$ 1B

1C $$\Phi =\frac{1-\kappa_{c}/\kappa_{m}}{3}+\frac{\rho_{c}/\rho_{m}-1}{2\rho_{c}/\rho_{m}+1}$$

where **e** *_y_* is the standard basis vector in the *y* direction. If the contrast factor Φ of a cell is positive, ***u***_r_ is directed toward the pressure node in the center of the channel, while for negative contrast, it is directed toward the nearest pressure antinode at the channel walls. Next, imagine an inhomogeneous fluid where the central region of the channel has lower κ_m_ and higher ρ_m_ than the regions on both sides. A cell initially near a wall may, in this scenario, at first be of positive Φ and move toward the pressure node in the channel center but will stop when reaching the transition region between positive and negative contrast.^33^ Likewise, if the cell starts near the center, it will move, due to negative Φ, to the same final location where Φ = 0. In this work, the central inhomogeneity consists of densely packed RBCs that we hypothesize can be described as a quasi-continuous medium that can alter the sign of the acoustic contrast of cells or other particles residing in the RBC fraction.

## Methods

### Chip and System

We used a glass-silicon-glass chip (GeSiM, Germany) with trifurcated in- and outlets (Figure 1A). The main channel dimensions are 375 μm in width, 150 μm in height, and 50 mm in length. The chip consists of a 0.50 mm thick bottom glass layer, a 0.76 mm top glass layer with access holes, and a silicon middle layer with the channel etched by deep reactive ion etching. The layers were bonded anodically. The inlets were connected to tubes through silicon tubes glued onto the chip. A piezoelectric transducer, glued to the side of the channel,^34^ driven by a function generator (33250A, Agilent), generated a sound field across the channel.

**Figure 1:**
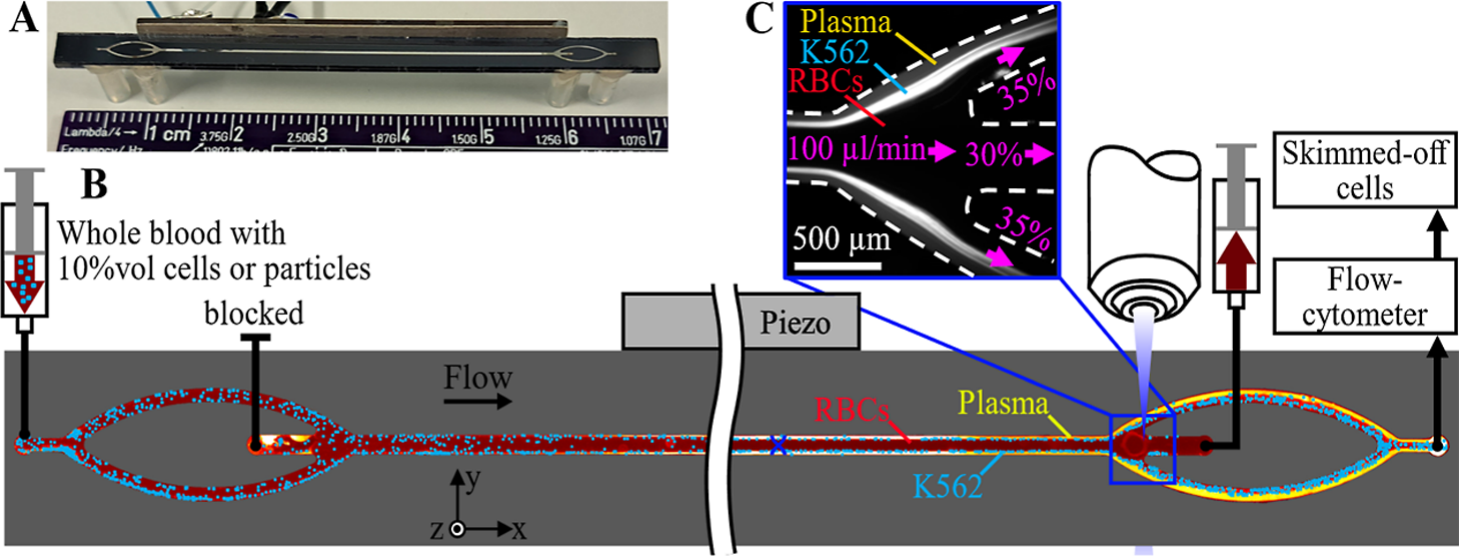
Chip and system. (A) Photo showing the chip with the etched channel and the piezoceramic transducer glued to one side. Fluid inlets are interfaced through pieces of silicone tubing glued to the back of the chip. (B) Schematic representation of the system and principle. (C) Inset showing the skimmed-off cells (bright) exiting through the side branches of the trifurcated outlet of the chip.

Blood was introduced into the chip through the side inlet using a syringe pump (Nemesys, Cetoni, Germany). The central inlet, used to prime the system with phosphate-buffered saline (PBS), was blocked during operation (Figure 1B).

Cell separation was monitored using an upright fluorescence microscope (BX51WI, Olympus) with a sensitive camera (Orca Flash 4, Hamamatsu). A LED light source with a liquid light guide (*p*E-4000, CoolLED) was used for fluorescence imaging, and a halogen lamp for bright field imaging.

### Sample Preparation

Blood was collected in EDTA tubes (BD Vacutainer, OneMed, Sweden) from healthy volunteers at Lund University (Lund, Sweden), with written informed consent, following the ethical principles of the Helsinki Declaration, through a procedure approved by the Swedish ethical review authority (ref no. 2020-05818).

K-562 cells (from Pereira lab, Division of Molecular Medicine and Gene Therapy, Lund University), a myelogenous leukemia cell line, were cultured in RPMI medium (Gibco, ThermoFisher) with 10% (v/v) fetal bovine serum (FBS, Sigma-Aldrich) and 1% (v/v) penicillins-streptomycin (Sigma-Aldrich) at 37 °C with 5% CO_2_. Cells were harvested, washed by centrifugation at 200 × RCF for 5 min, and resuspended in PBS. To fluorescently stain the cells, 25 μM Green CMFDA (ThermoFisher) was added to the cell suspension (ca. 2 million cells/mL) and incubated for 30 min at 37 °C. After incubation, cells were washed twice by centrifugation at 200 × RCF for 5 min and resuspension in PBS. The stained cell suspension was mixed with whole blood, diluting the blood sample to 90% (v/v). For experiments with microparticles, blood was spiked with 10% (v/v) of red fluorescent 48.2 μm Polystyrene (PS) (PS-FluoRed-50, Microparticles GmbH, Germany), red fluorescent 9.98 μm PS (PS-FluoRed-10.0, Microparticles GmbH, Germany), or green fluorescent 5.0 μm PS (Fluoro-Max G0500B, Thermo Fisher Scientific) particles in PBS.

White blood cell (WBC) subpopulations, lymphocytes, neutrophils, and monocytes were isolated by negative selection using EasySep Direct Human Isolation Kits (STEMCELL Technologies, Canada) for each cell type, following the manufacturer’s protocol. The isolated white blood cells were centrifuged at 200 × RCF for 5 min, resuspended in PBS, and stained as described above for K-562 cells.

### Imaging Cell and Particle Separation

We imaged the localization of cells and particles with different acoustic properties after acoustic packing during flow. Suspensions of either stained K-562 cells, WBCs (monocytes, lymphocytes, neutrophils) or PS microparticles were mixed with whole blood to a final whole blood volume fraction of 90% (v/v) and injected into the chip at a flow rate of 100 μL min^–1^, unless otherwise stated. A standing acoustic wave was formed in the channel by piezo actuation at a frequency of 1.997 MHz and a voltage *U*_pp_ of 10.0 V. Image series (5 × 500 images at 10 ms exposure time) were captured 2 mm upstream of the outlet trifurcation for each fluorescent cell or particle type. Brightfield images (500 images, 10 ms exposure time) were recorded to localize the RBC–plasma interface and channel walls.

Channel wall and blood–plasma interface coordinates were determined from each brightfield image using adaptive thresholding within defined subregions, followed by calculating the temporal median across frames and Gaussian smoothing for robustness. A background image was generated by computing the temporal median of every 10th frame from fluorescence image series. The background was subtracted from all fluorescence imaging frames, after which a time-averaged image of the corrected frames was computed, denoised using median filtering, and normalized to its maximum pixel intensity. The fluorescence intensity across the *y*-axis was calculated by averaging this image along the *x*-axis and then normalizing the result.

### Flow Cytometry Cell and Particle Counting

Samples from side and center outlets were collected for all cells and particles, except the 50 μm PS particles, at a flow rate (*Q*) of 100 μL min^–1^ and a center/total flow rate ratio of 50%. All samples were diluted 1:1000 with PBS and the cell concentration (*c*) was analyzed using flow cytometry (Cytoflex, Beckman Coulter. Figure S1). Control experiments without acoustic actuation were performed for all particles and cells.

### Cancer Cell Enrichment

To demonstrate cancer cell enrichment from blood, a mixture of 90% (v/v) whole blood and a 10% (v/v) K-562 cell suspension stained with CellTracker Green CMFDA was perfused through the chip via the side inlets using a 1 mL syringe pump at a flow rate of 100 μL min^–1^. The center inlet was blocked. Best cell focusing was found at a piezo actuation frequency of 1.940 MHz. Samples were collected from a tube, open to atmospheric pressure, connected to either the center or the side outlet while the other outlet was connected to a syringe pump running in aspiration mode to control the output flow rates. A 50 μL sample was collected for each central outlet flow-split ratio (30%, 40%, 50%, 60%, 70%, and 80% of the total). Samples were diluted 1:1000 with PBS and 120 μL of each diluted sample was analyzed using a flow cytometer with absolute volume measurement to quantify RBCs and K-562 cells in every sample. Fluorescence images of the outlet trifurcation were captured for each flow ratio. Samples from three different donors were analyzed in triplicate. The HCT of every sample was measured by centrifugation in HCT capillaries. The stated HCT was calculated for the 90% (v/v) mixture. The separation parameters RBC center fraction, K-562 side fraction, and K-562 relative enrichment in the side outlet sample were defined as

$$K‐562\;side\;fraction=\frac{c_{K562,side}Q_{side}}{c_{K562,side}Q_{side}+c_{K562,center}Q_{center}}$$ 2A

2B $$RBC\;center\;fraction=\frac{c_{RBC,center}Q_{center}}{c_{RBC,center}Q_{center}+c_{RBC,side}Q_{side}}$$

2C $$K‐562\;relative\;enrichment=\frac{c_{K562,side}}{c_{RBC,side}}\cdot \frac{c_{RBC,side}Q_{side}+c_{RBC,center}Q_{center}}{c_{K562,side}Q_{side}+c_{K562,center}Q_{center}}$$

### Cancer Cell Skimming at Different Flow Rates

We examined cancer cell separation from blood for different flow rates for a mixture of 90% (v/v) whole blood and 10% (v/v) K-562 cell suspension stained with CellTracker Green CMFDA. The cell suspension was perfused through the chip via the side inlets using a 1 mL syringe in a pump at flow rates 100, 250, 300, 350, 400, and 500 μL min^–1^. The outlet flow-split ratio was set to 50%. The piezo actuation frequency was 1.952 MHz and the power to the piezo was 70.05 mW. Five times 300 frames (10 ms exposure time) were recorded for every flow rate. The images were averaged over time and data sets and normalized. For every flow rate, 50 μL triplicate samples were collected from the center and side outlet, respectively. The samples collected were then diluted 1:1000 with PBS and analyzed by flow cytometry.

### Acoustic Cell Focusing at Different Hematocrit

Blood-K-562 suspensions with different HCT (vol % of cells in blood after centrifugation) were created by diluting whole blood with the stained K-562 suspension and PBS or by centrifuging the blood (10,000 × RCF for 5 min) and removing fractions of the plasma before adding the K-562 cell suspension. Each suspension was driven through the chip at a flow rate of 100 μL min^–1^, with a forced split ratio of 50% at the center and side outlet. The piezo actuation frequency was adjusted so that maximal possible focusing was achieved. 100 images (10 ms exposure time) were recorded for each HCT, then time-averaged, and normalized.

## Results

### Cell Locations during Flow

We studied the self-organization after acoustic packing of crowded cell suspensions by spiking fluorescently labeled K-562 cells or WBC subgroups into whole blood and driving the suspension through an acoustophoretic channel at a constant flow rate. The positions of the spiked cells relative to the RBC–plasma interface were recorded near the channel outlet (Figure 2A). As previously reported, the acoustic radiation force moves the RBCs to the center of the channel, leaving cell-free plasma at the sides.^28,31,35^ The K-562 cells can be observed to localize at the RBC–plasma interface (Figure 2B) indicating that they are expelled from the packed RBCs due to having negative acoustic contrast. Since optical localization using fluorescence microscopy provides an incomplete representation of cell positions due to scattering and absorption of light in the packed RBCs, we quantified the final location of cells and particles in the two outlets of the chip by flow cytometry. Figure 2B shows that 98% of the K-562 cells end up in the side fraction, confirming the distribution shown in the fluorescence image. Monocytes (Figure 2C) and lymphocytes (Figure 2D) have similar distributions as K-562 cells, confirmed by the flow cytometry data. While the neutrophil distribution shows a dip in fluorescence intensity in the middle of the channel, the flow cytometric quantification shows the same relative distributions of blood cells and neutrophils in the center and side fractions (Figure 2E). PS microparticles exhibit localizations indicative of positive acoustic contrast in packed RBCs due to their relatively lower compressibility.^36–38^ Larger 48.2 μm PS particles concentrate at the center of the packed blood cells (Figure 2F). The 9.98 μm PS microparticles are also localized at the center, albeit with a broader *y*-spread (Figure 2G), and flow cytometry reveals that almost all of them are routed to the center outlet. The 5.0 μm PS microparticles were distributed across the whole width of the packed RBCs with an increased bimodal concentration off-center, close to the two RBC–plasma interfaces (Figure 2H). This may be an optical artifact, originating from less obstructed particles at the blood–plasma interface, or they may be slow to penetrate the packed RBCs. Flow cytometer analysis of the 5.0 μm PS output samples (Figure 2H) reveal a slightly higher concentration of particles in the center outlet. No-sound controls for all particles and WBCs cells showed elevated fluorescence intensities near the channel walls (Figure S2), possibly due to margination or other effects, but they are distinct from the distributions caused by acoustic actuation.

**Figure 2:**
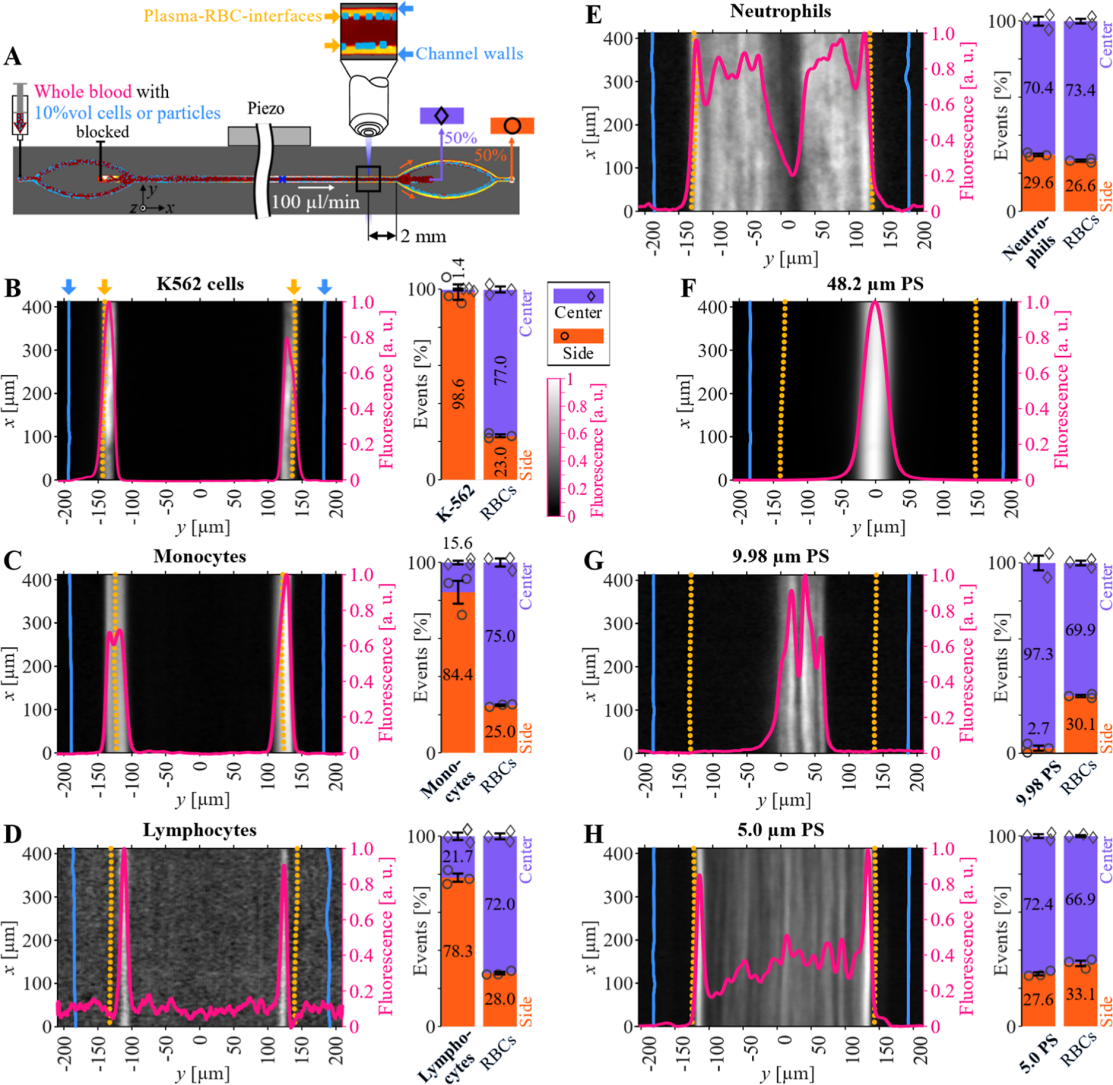
Localization of cells and particles after acoustic packing in blood. (A) Experiment schematic with chip setup and imaging window 2 mm upstream of the outlet trifurcation. Intensity distributions across the width of the channel in the packed RBC region and flow cytometric quantification of cells from center and side output samples at a center-to-side split of 50:50 for (B) K-562 cells, (C) monocytes, (D) lymphocytes, (E) neutrophils, and for PS microparticles of the sizes (F) 48.2 μm, (G) 9.98 μm, and (H) 5.0 μm. Dotted yellow line marks the RBC–plasma interfaces and continuous blue line marks the channel walls.

### Flow-through Enrichment of Rare Cells from Whole Blood

To investigate whether subgroups of cells can be enriched through cell self-organization in an acoustic field, we configured the system for in-line processing. By adjusting the relative flow rate between the central and side outlets, we controlled the separation of different proportions of spiked cells and RBCs into their respective outlets (Figure 3A and Video S1). For a flow rate split ratio of 30% (center outlet flow rate/total flow rate), >98% of K-562 cells can be recovered (Figure 3A,B), as they are far enough separated from the center outlet by packed RBCs. As the split ratio increases, fewer RBCs are skimmed off and exit through the center outlet causing the RBC–plasma interface, and consequently, the K-562 cells, to move inward. At a 60% split ratio, the interface starts splitting between the side and center outlet channel, leading to a significant drop in the K-562 side-fraction. At a split ratio of 80% most K-562 cells exit through the center outlet. The RBC center fraction at a split ratio of 30% is about 45% and increases as the split ratio rises, reaching almost 100% at a split ratio of 70% (Figure 3C), meaning most RBCs exit through the center outlet. K-562 relative enrichment is maximum 14 to 43-fold, depending on the donor HCT, at a flow-split ratio of 70% (Figure 3D). Noticeably, donor 3 (48% HCT) shows, especially at a split ratio of 70–80%, a slightly higher K-562 side-fraction and lower RBC center fraction compared to other donors with lower HCT (40% HCT). As platelets were conservatively counted within the RBC fraction (Figure S1), their increased fraction in the side outlet at higher flow-split ratios (60–70%), influences the measured K-562 enrichment values (Figure S3), which can reach over 100 fold enrichment relative to only RBCs.

**Figure 3:**
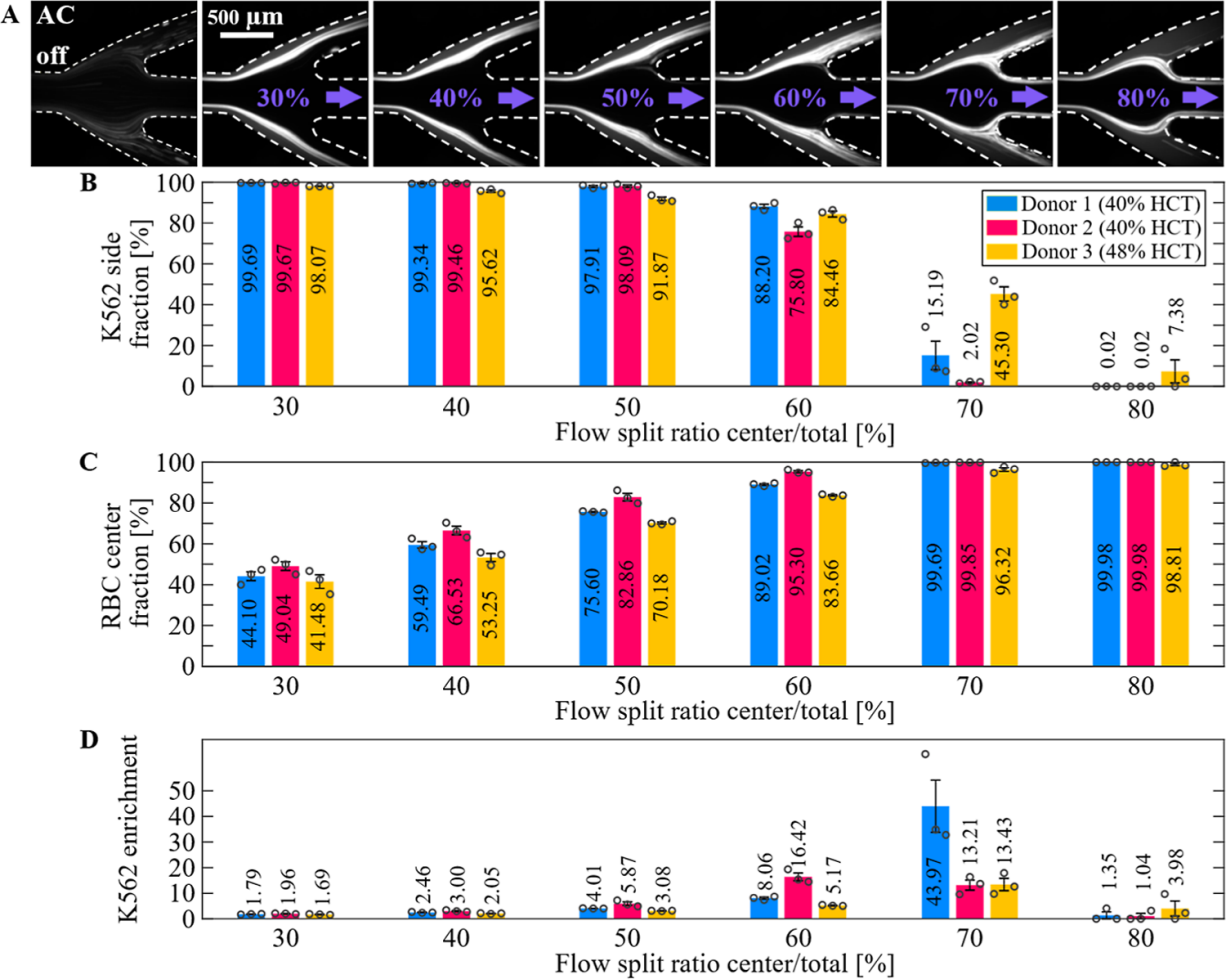
Flow-through enrichment of K-562 cells from whole blood using acoustic packing and skimming. (A) Photos of the outlet trifurcation when varying the center-to-total volume flow-split ratio. Triplicate samples from three different donors for different flow-split ratios were analyzed by flow cytometry showing (B) K-562 cell side-fraction, (C) RBC center fraction, and (D) K-562 cell enrichment relative to RBCs plus PLTs in the side outlet.

### Flow-through Focusing at Higher Flow Rates

To examine the influence of higher flow rates on the performance of our cell separation, we carried out a skimming experiment with varying flow rates at a fixed 50% outlet flow ratio. At a flow rate of 100 μL min^–1^, the RBC–plasma border, and the fluorescent K-562 cells located there, are skimmed off with a generous margin of RBCs (Figure 4A). 99% of fluorescent K-562 cells were collected at the side outlet (Figure 4D), while 77% of RBCs were found at the center outlet (compare Figure 3). With the increasing flow rate, the band of focused K-562-cells first moves closer to the outer walls of the side channel and becomes fuzzier (Figure 4B,C). The collected cell samples show a relatively constant fraction of over 95% of skimmed fluorescent cells at the side outlet (Figure 4D) until 300 μL min^–1^, where this fraction falls off under 90%. At the same time, the number of skimmed-off RBCs in the side fraction increases from 23% at 100 μL min^–1^ to 38% at 500 μL min^–1^. The acoustic packing and flow were not stable under 100 μL min^–1^.

**Figure 4:**
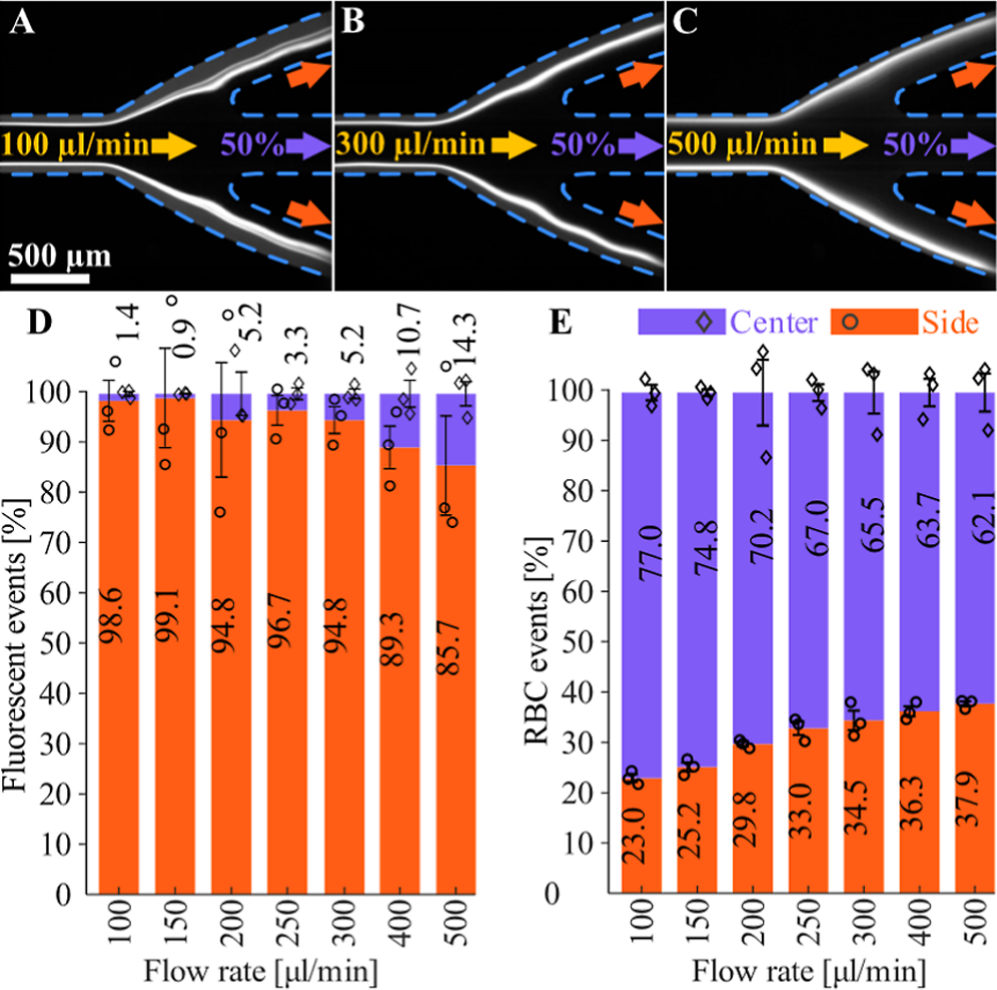
Effect of flow rate on K-562 cell skimming and RBC outlet fractions. (A–C) Fluorescence images of K-562 cell localization at the output trifurcation at flow rates of 100, 300, and 500 μL min^–1^. Blue dashed lines highlights the channel walls. (D) Counted fluorescent cells from side and center outlet. (E) RBCs counted from side and center outlet samples.

### Effect of Hematocrit

To investigate the influence of HCT on the ability to relocate K-562 cells to the RBC–plasma interface, we made samples of different HCT by removing or adding plasma to the blood. Figure 5A–F shows that a layer of K-562 forms at the interface for all investigated HCTs. The location of the RBC–plasma interface depends linearly on the HCT^31^ which implies that to achieve successful separation, the flow-split ratio must be tailored to the HCT by e.g. adjusting the flow-split ratio.

**Figure 5:**
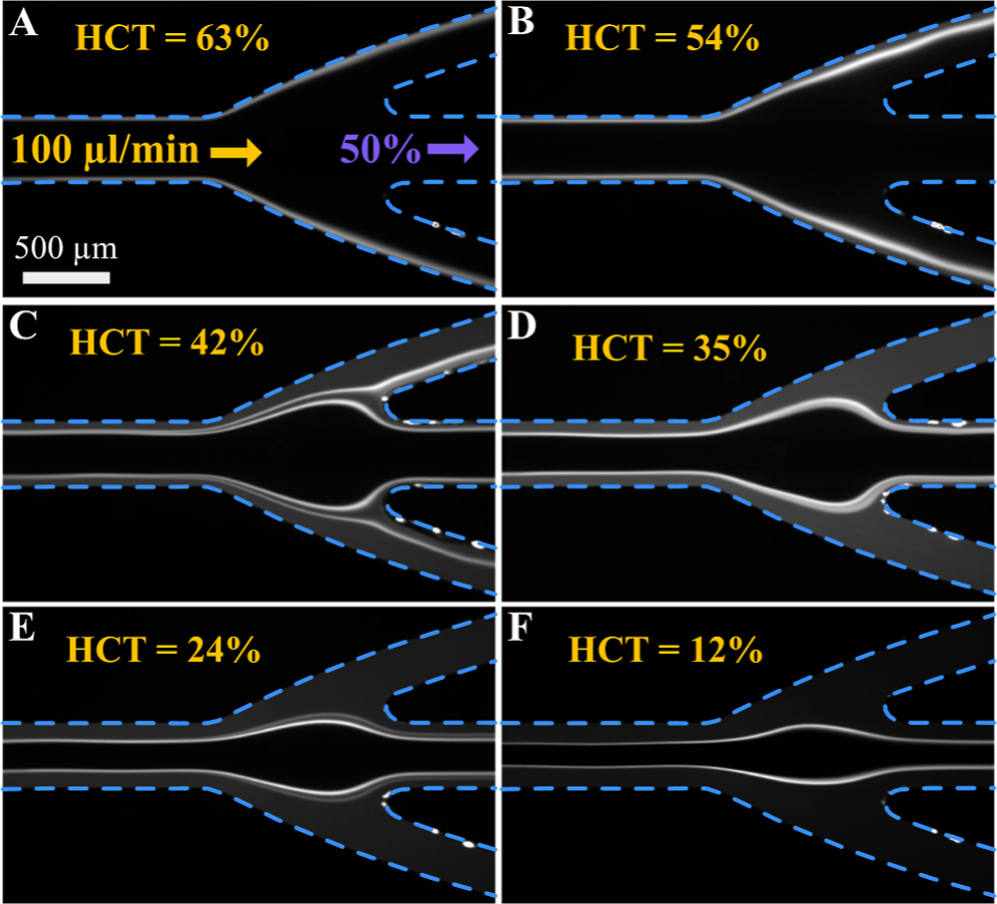
Effect of hematocrit. (A–F) Fluorescence images of K-562 cell localization at the output trifurcation for hematocrits decreasing from 63% to 12%. Blue dashed lines highlight the channel walls.

## Discussion

Blood is a complex and dense suspension of cells that consists primarily of RBCs (∼44%), WBCs (<0.1%), platelets (<1%), and blood plasma (∼55%). This complexity, and the nature of cells as deformable and slippery particles, make hydrodynamic and acoustic modeling of dense cell suspension very challenging. In the absence of a complete and verified theoretical model, we make the following assumptions: As RBCs become more packed, the cell–cell interactions become frequent, and each cell will be surrounded by RBCs in full contact. Any particle or cell, surrounded by this quasi-continuous RBC medium will not be defined by the cell-to-plasma mass density and compressibility, but rather by the cell-to-RBC contrast. The acoustic properties of plasma are close to those of water and we hypothesize that PS particles, RBCs, WBCs, and K-562 cells have a positive contrast factor in plasma and can be arranged in the following order: Φ_PS,plasma_ > Φ_RBC,plasma_ ≥ Φ_WBC,plasma_ ≥ Φ_K‑562,plasma_ > 0.^33,36–42^ Hence, in an acoustic standing wave field, all the cells and particles examined here move toward the pressure node in the channel center at different velocities, depending on their acoustic contrast factor and size (eqs 1A,B). Once cells are packed, their surrounding medium is no longer plasma, but the quasi-continuous RBC medium and their acoustic contrast factor will depend on their acoustic properties relative to the packed RBCs such that all cells except neutrophils have positive contrast (Video S2). The negative contrast of K-562 cells, monocytes, and lymphocytes in packed RBCs leads to their migration toward the pressure antinodes at the channel wall but once reaching the RBC–plasma interface they stop due to a positive contrast factor in plasma (Figure 2B–D). While the compressibility of neutrophils is unknown, their density is close to that of RBCs, indicating that their acoustic contrast factor in packed RBCs may be close to 0, preventing them from migrating to the RBC–plasma interface (Figure 2E). The dip in the fluorescence intensity distribution hints that they may have a slightly negative contrast factor in packed RBCs and that enrichment could be possible for higher acoustic amplitudes. PS particles on the other hand have a positive contrast factor in plasma as well as in packed RBCs and their tendency to focus in the channel center increases with size (Figure 2F–H). The 5 μm PS particles had a similar distribution as for a no-sound condition (Figures 2H and S2H). We think this is due to the high drag force on the particles due to the exponentially increasing viscosity of blood as a function of HCT.^43^ This leads to a considerably longer time to focus, and particles may in addition be affected by acoustic streaming inside the packed RBCs.

Blood is an extremely complex suspension, and our simplistic model does not capture the dynamics of the acoustic cell reorganization. First, in the transient phase, cells are surrounded primarily by plasma and the RBC concentration then increases gradually as the RBCs pack together. Second, cells in close vicinity of other cells experience hydrodynamic coupling to each other^27^ and the sound field itself may also be strongly affected during the reorganization. Third, while flowing through the channel cells may additionally be subject to margination^44–46^ and shear-induced diffusion^44–46^ which can also affect the separation. The ability to pack RBCs in the central outlet decreases with an increasing flow rate (Figure 4A–C). Despite the observed outward displacement of the fluorescence intensity from the skimmed cells, the flow cytometry analysis reveals that the ability to recover K-562 cells through the side outlet decreases with flow rate and the loss escalates at flow rates exceeding 300 μL min^–1^ (Figure 4D). This indicates that the cells do not have sufficient time to completely self-organize before reaching the end of the channel or that the looser packing of the RBCs due to shear-induced diffusion^29^ lead to less efficient self-organization.

The ability to enrich cancer cells is a compromise between RBC depletion and K-562 cell recovery. Cancer cell relative enrichment was highest (13 to 44 times, Figure 3D) at a center-to-total outlet flow-split ratio of 70%. At this and higher flow-split ratios, the RBC side fraction reaches almost 100%, while at split ratios lower than 50%, the cancer cell fraction reaches almost 100% (Figure 3B,C). This suggests that most K-562 cells are at the RBC–plasma interface and that the packed RBCs are almost void of K-562 cells. Lymphocytes and monocytes show similar trends as cancer cells while neutrophils remain among the packed RBCs after separation (Figure 2B–E).

We show that ∼90% of RBCs can be depleted while recovering >74% of the target cancer cells. This is comparable in performance to a so-called buffy coat preparation by a blood bank centrifuge that has a mononuclear cell recovery of around 80%, and with 80% depletion of RBCs^47^ and for leukapheresis, 95% depletion of RBCs has been reported.^48^ Traditional Ficoll gradient methods typically achieve up to 99% RBC depletion,^49^ and automated systems like the Sepax-2 can increase this to 99.8% while typically recovering 78–85% of mononuclear cells.^49–51^

Blood HCT, but also blood viscosity varies between individuals and their status. Figure 5 shows that the HCT impacts separation, and we think the performance of our method can be drastically improved by dynamic adaptation of the operating parameters. This can be achieved by image-based surveillance and automated tuning of the outlet streams. Unlike immunomagnetic separation, our method cannot differentiate WBCs from cancer cells when they have similar acoustic properties. So, this method may be best used as an initial enrichment stage, coupled with post-processing with other acoustofluidic^24,33^ or microfluidic^11,15,52^ methods. Another possibility for improvement would be to further separate the RBCs from the cells of interest, using for example a barrier medium like iodixanol.^53^

## Conclusions

We have demonstrated a novel acoustofluidic method for the enrichment of cancer cells directly from whole blood by exploiting cell self-organization in acoustic fields. We have proposed a simple model for the self-arrangement of the blood cells based on differences in acoustic contrast between different cell types when surrounded by packed RBCs. K-562 cancer cells and mononuclear cells could be continuously skimmed off at the RBC–plasma interface, achieving efficient RBC-depletion and cancer cell recovery. We explored the limits of the method at higher flow rates and high HCTs. The method can serve as an initial enrichment step in a series of purification processes for accessing rare cells directly from blood in a format that has the potential for system integration. Future work will focus on better understanding the complex fluid dynamics of the separation, optimizing the system for individual samples, and integrating additional separation stages to enhance specificity and throughput.

## Supplementary Material







## Notes

Video of separation at outlet trifurcation (MP4)
Video of acoustic focusing of blood and cell at stopped flow (MP4)
Flow cytometer gating for classification and enumeration of separated cells and cell localizations for no-sound experiments. Analysis is subdivided by PLT and RBC fractions (PDF)
