# Supplementary material for: In-Line Enrichment of Cancer Cells from Whole Blood by Cell Self-Organization in Acoustic Fields
Source: Anal Chem. 2025 Jun 14;97(25):13310–7. doi: 10.1021/acs.analchem.5c01459 (PMC12224171; doi:10.1021/acs.analchem.5c01459)
Supplement: Supplementary file 3 [file ac5c01459_si_003.pdf]

# SUPPORTING INFORMATION

**In-line enrichment of cancer cells from whole blood by cell self-organization in acoustic fields.**

Richard Soller, Ola Jakobsson, and Per Augustsson*

Lund University, Department of Biomedical Engineering, 223 63, Lund, Sweden
* Corresponding author, e-mail: per.augustsson@bme.lth.se
URL: https://bme.lth.se/english/research/nanobiotechnology-and-lab-on-a-chip

**Table of Contents** **Page**

| | | |
|---|---|---|
| S1 | Flow cytometer gating examples | s2 |
| S2 | Cell localizations for no-sound condition | s3 |
| S3 | RBC and PLT fractions in the side outlet. RBC and PLT center fractions. K-562 enrichment relative to only RBCs or PLTs | s4 |
| S4 | Supporting video 1 description | s5 |
| S5 | Supporting video 2 description | s6 |

## S1. Flow cytometer gating – separated cells

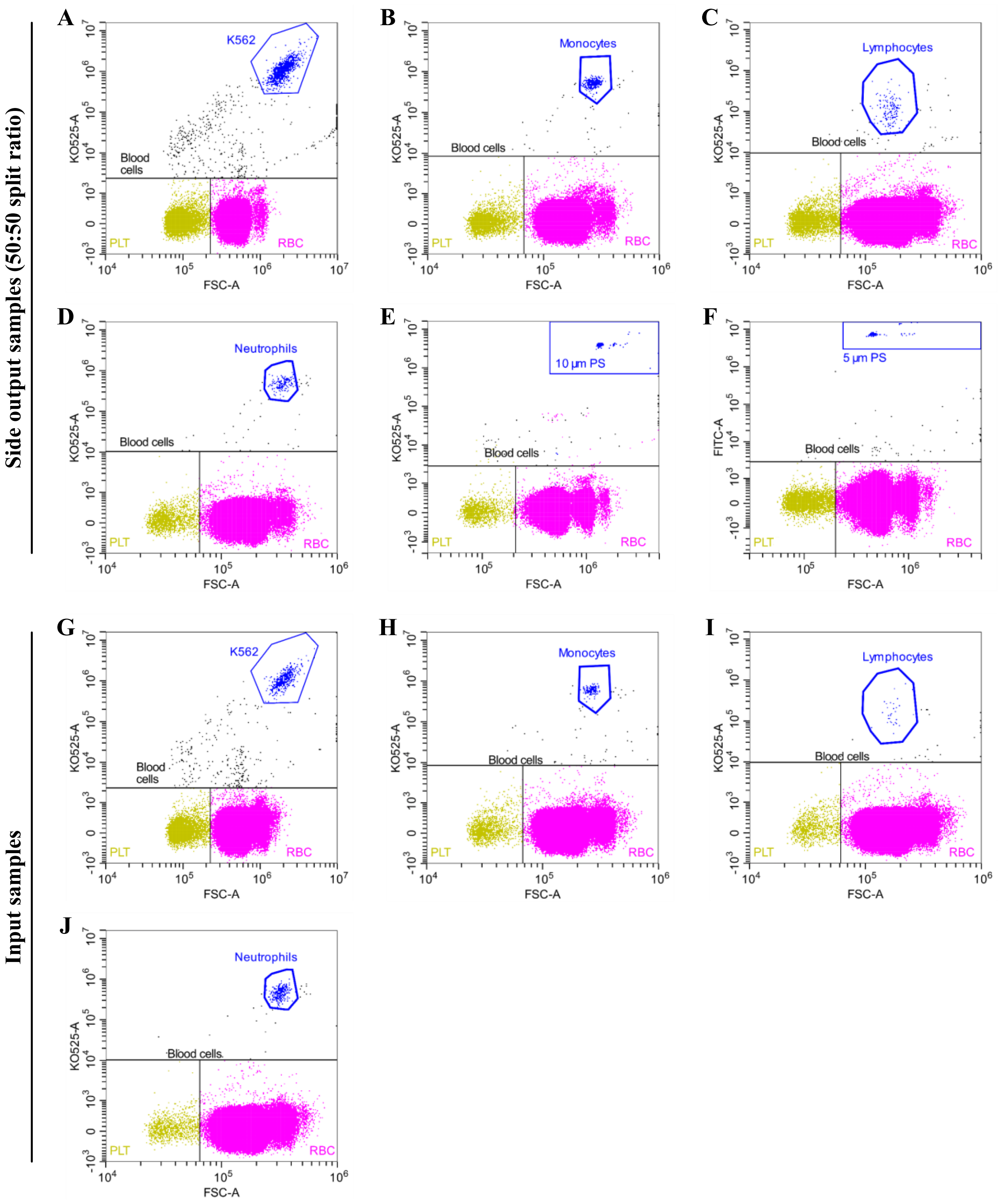


**Figure S1:** Flow cytometry gating examples showing a magenta gate for red blood cells (RBCs) and a yellow gate for platelets (PLTs), which together constitute the blood cell population. Since platelets are not labelled – to be conservative – for our calculations, we equate the RBC population with the blood cell population. White blood (WBC) cells innate in the blood sample are included in the RBC population and neglected. Cells or particles of interest are identified by the blue gate. Flow cytometry dot plots for side output samples (50:50 flow split ratio at output trifurcation) for stained (A) K-562 cells, (B) monocytes, (C) lymphocytes, and (D) neutrophils, as well as polystyrene (PS) particles of sizes (E) 10 µm and (F) 5 µm. Plots for input samples for stained (G) K-562 cells, (H) monocytes, (I) lymphocytes, and (J) neutrophils.

## S2. Cell localizations for no-sound condition

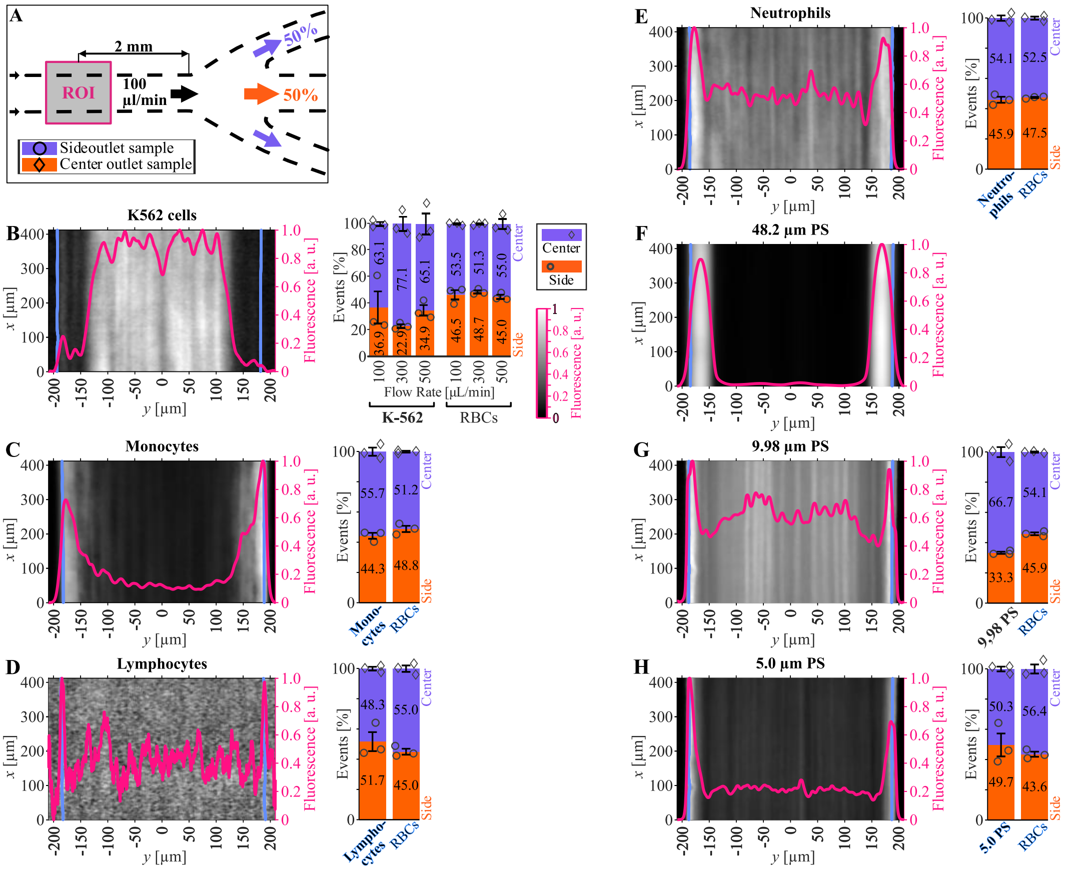

**Figure S3:** Localization of cells and particles after passing the channel without acoustic packing. (A) Experiment schematic with imaging window 2 mm upstream of the outlet trifurcation. From the images were determined the distributions across the width of the channel (*y*), delimited by blue vertical lines, of (B) K-562 cells, (C) monocytes (Mono), (D) lymphocytes (LYM), (E) neutrophils (NEU), and for polystyrene (PS) microparticles of the sizes (F) 48.2 µm, (G) 9.98 µm, and (H) 5.0 µm in the RBC region. Next to each *y*-distribution graph, flow cytometric quantification is presented for (B) K562 cells at flow rates of 100, 300, and 500 µl/min, and at 100 µl/min for (C) monocytes, (D) lymphocytes, (E) neutrophils, (G) 9.98-µm PS particles, (H) 5.0-µm PS particles and RBCs. The measurements were taken from center and side output samples at a 50:50 split ratio.

## S3. RBC and PLT fractions in the side outlet. RBC and PLT center fractions. K-562 enrichment relative to only RBCs or PLTs

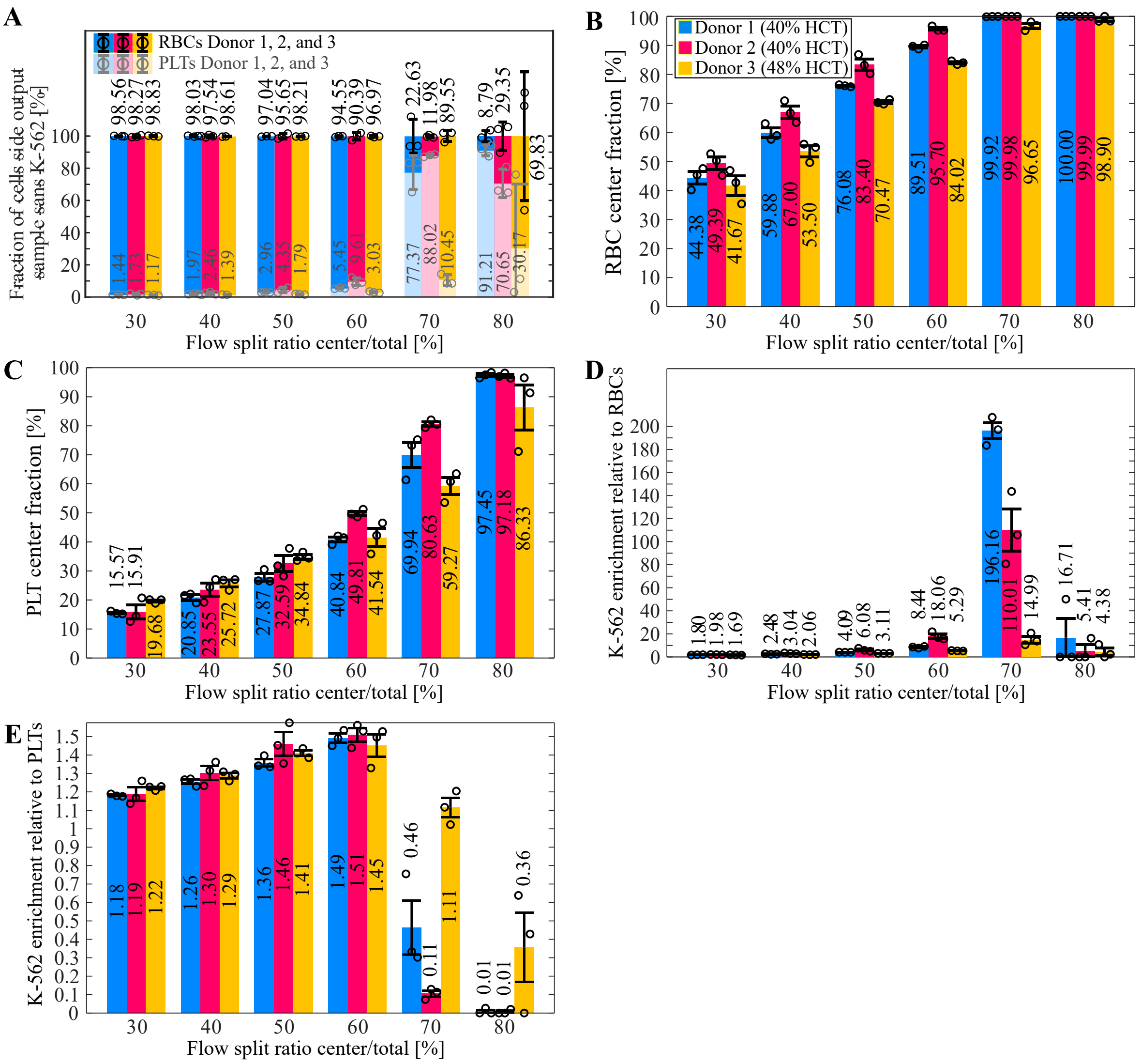


**Figure S3**: The data shown in Figure 3 includes PLTs in the RBC population as explained in Figure S1. (A) The composition of the side output sample sans K-562 cells. It consists mostly of RBCs with a slowly growing share of PLTs. Between a flow split ratio of 60 and 70%, the fraction of PLTs suddenly increases over the fraction of RBCs as most of the RBCs exit through the center outlet while acoustically not focused PLTs in the plasma exit though the side outlets. (B) The RBC-only center fraction and (C) the PLT-only center fraction. (D) The enrichment of K-562 cells relative to only the RBC. Especially for the lower HCT samples, the RBC-only enrichment at a flow split ratio of 70% is much higher than the enrichment relative to RBC with PLT. (E) The enrichment of K562-cells relative to only PLTs. The K-562 enrichment relative to PLTs only increases slowly with increasing flow split ratio as the fraction of PLTs in the side outlet increases too. At a split ratio of 70%, the K-562 fraction in the side outlet sharply decreases and the enrichment of K-562 cells relative to PLTs drops too.

## S4. Supporting video 1 description

**Filename:** Supporting_video_1.mp4

**Supporting Video 1:** Monocytes skimmed off from blood at the output trifurcation at a 100 μl/min flow rate and an output split ratio of 50:50. Brightness and contrast were adjusted with 1% saturation at low and high ends of the intensity histogram. Gamma correction was applied with a gamma value of 0.5.

## S5. Supporting video 2 description

**Filename:** Supporting_video_2.mp4

**Supporting Video 2:** K-562 cells acoustically focused after flow is stopped. 90% (v/v) whole blood and 10% (v/v) K-562 cell suspension. Brightness and contrast were adjusted with 0.1% saturation at low and high ends of the intensity histogram. Gamma correction was applied with a gamma value of 0.5. The K-562 cell first focuses, together with the RBCs, towards the channel center. After RBCs are packed more densely, the K-562 cell stops going to the center and moves to the RBC-Plasma interface.
